# The Role of Robotic-Assisted Surgery in Orthopedic Practice: A Comprehensive Review of Opportunities, Challenges, and Future Directions

**DOI:** 10.7759/cureus.92337

**Published:** 2025-09-15

**Authors:** Ahmed Elkohail, Ahmed M Khalifa, Ali Soffar, Ibrahim Omar, Mostafa Abdulaziz, Ahmed Elsaket, Ahmed Elnewishy, Momen Abdelglil, Siddhartha Murhekar, Ahmed Swealem

**Affiliations:** 1 Orthopaedics and Trauma, Princess Royal University Hospital, King's College Hospital NHS Foundation Trust, London, GBR; 2 Cardiology, Frimley Park Hospital, Frimley Health NHS Foundation Trust, Frimley, GBR; 3 Trauma and Orthopaedic Surgery, Princess Royal University Hospital, King's College Hospital NHS Foundation Trust, London, GBR; 4 Urology, Wrexham Maelor Hospital, Wrexham, GBR; 5 Cardiology, University Hospital Monklands, Lanarkshire, GBR; 6 Orthopaedics, Frimley Health NHS Foundation Trust, Camberley, GBR; 7 Trauma and Orthopaedics, Royal Berkshire Hospital, Reading, GBR; 8 Pediatric Surgery, Mansoura University Children's Hospital, Mansoura, EGY; 9 Trauma and Orthopaedics, Medway NHS Foundation Trust, Gillingham, GBR; 10 Orthopaedics, North Bristol NHS Trust, Bristol, GBR

**Keywords:** clinical outcomes, future perspectives in orthopedic robotics, orthopedic surgery, robotic-assisted surgery, robotics, surgical precision

## Abstract

Robotic-assisted surgery has emerged as an important innovation in orthopedic practice, offering millimeter-level operative accuracy, enhanced reproducibility, and the potential for improved clinical outcomes. Its applications span joint replacement, spine surgery, trauma fixation, and expanding areas such as oncology and complex reconstructions. Evidence demonstrates superior implant alignment, reduced radiation exposure, and improved perioperative outcomes compared to conventional techniques; however, the long-term functional benefits and cost-effectiveness remain debated. Recent advances, including artificial intelligence, optical navigation, haptic technology, and augmented reality, are expanding the scope and accuracy of robotic platforms. Despite promising outcomes, challenges such as high costs, steep learning curves, and limited accessibility persist, hindering widespread adoption. Future integration of AI-driven decision support, real-time imaging, and more autonomous tactile systems is expected to further personalize and refine orthopedic care. This review provides a comprehensive synthesis of current applications, technological innovations, limitations, and future directions of robotic-assisted orthopedic surgery.

## Introduction and background

Robotic-assisted surgery has rapidly transformed orthopedic practice, offering enhanced precision, reproducibility, and potential improvements in patient outcomes across a range of subspecialties, including joint arthroplasty, spine, trauma, and emerging areas like foot, ankle, and shoulder surgery [1-4]. The adoption of robotic systems has increased steadily, particularly in high-volume centers and among surgeons with access to advanced technology [5-7].

Robotic technology provides millimeter-level precision, reduces human error, and facilitates personalized, minimally invasive interventions. The evolution from the early ROBODOC system in 1992 to modern platforms such as MAKO and ROSA reflects the steady integration of robotics, especially in joint replacement and spinal surgery [8]. Comparative studies reveal that robotic-assisted orthopedic surgeries achieve superior precision and faster recovery compared to conventional techniques, showing measurable gains in alignment accuracy and overall postoperative outcomes [9-15].

The integration of artificial intelligence (AI), augmented reality, and haptic feedback is anticipated to further enhance the capabilities and accessibility of robotic systems in orthopedics [16-18]. As the field evolves, ongoing research is needed to clarify the impact of robotic assistance on long-term outcomes, cost-effectiveness, and broader applications [4,19-23].

Despite challenges such as high costs, steep learning curves, and limited long-term data, ongoing technological advancements and expanding clinical indications continue to drive the integration of robotics into mainstream orthopedic care [24-27]. This review synthesizes the current landscape, clinical applications, outcomes, and future directions of robotic-assisted orthopedic surgery.

## Review

Methodology

This review was conducted as a comprehensive narrative synthesis of the literature on robotic-assisted surgery in orthopedic practice. A narrative approach was employed to ensure breadth and depth of coverage across clinical applications, recent technological advances, limitations, and future directions.

Relevant studies were identified through an extensive search of electronic databases, including PubMed, Scopus, Web of Science, and Google Scholar, using combinations of the following keywords: “robotic-assisted surgery”, “orthopedic surgery”, “robotics in orthopedics”, “robotic joint replacement”, “robotic spine surgery”, “robotic trauma surgery”, and “future perspectives in orthopedic robotics”. The search encompassed publications from the early introduction of orthopedic robotics in the 1990s up to January 2025.

Original clinical studies, systematic reviews, meta-analyses, and high-quality narrative reviews focusing on robotic-assisted orthopedic surgery were included. Articles addressing clinical applications, outcomes, safety, cost-effectiveness, and technological innovations were also included. Exclusion criteria included conference abstracts without full manuscripts, non-peer-reviewed commentaries, and studies unrelated to orthopedic surgical applications of robotics.

To ensure reliability, the references of included studies were screened manually to capture additional relevant publications not identified in the initial search. Extracted data were synthesized thematically into key domains: clinical applications (arthroplasty, spine, trauma, and oncology), technological innovations (AI integration, imaging, navigation, and haptics), limitations and barriers, and future directions.

Given the narrative and comprehensive scope of this review, no meta-analysis or quantitative pooling of outcomes was performed. Instead, emphasis was placed on highlighting trends, summarizing high-level evidence, and identifying research gaps to guide future investigations.

Clinical applications of robotic-assisted orthopedic surgery

Orthopedic surgical robotics can be grouped into three categories based on automation and control. Autonomous systems operate independently once initiated. Semi-autonomous systems allow surgeon control with robotic assistance, offering guidance and precision. Teleoperated systems use master-slave configurations, where the surgeon controls instruments via a console, gaining precision and dexterity [25].

This classification framework further extends to distinguish between image-based systems that require preoperative CT or MRI for surgical planning and imageless systems that utilize intraoperative data acquisition through anatomical landmark registration. Additionally, systems are categorized as active when they perform direct bone cutting, passive when they provide guidance only, and semi-active when they offer controlled assistance with haptic boundaries. The degree of automation ranges from fully manual surgeon control to complete robotic autonomy, with most contemporary systems operating in the semi-autonomous realm to optimize both surgical precision and surgeon expertise [16].

Robotic-Assisted Joint Replacement Surgeries

Robotic surgery has rapidly transformed the landscape of joint replacement, particularly in total knee arthroplasty (TKA), total hip arthroplasty (THA), and unicompartmental knee arthroplasty (UKA). Over the past two decades, robotic-assisted systems have been developed to enhance surgical precision, improve implant alignment, and potentially optimize patient outcomes [25,28-31]. Robotic systems in joint replacement have evolved from early active systems (e.g., ROBODOC) to current semi-active and haptic-guided platforms (e.g., MAKO and Navio) [25,32,33].

Evidence consistently demonstrates that robotic systems improve the accuracy and reproducibility of implant positioning and alignment compared to conventional manual techniques [2,29,34,35]. Some studies report early benefits such as reduced postoperative pain, faster functional recovery, and higher patient satisfaction, especially in UKA and early postoperative periods [15,36,37]. Robotic-assisted arthroplasty consistently demonstrates superior accuracy in implant positioning, alignment, and restoration of joint biomechanics compared to manual techniques [2,29,31,32,34,35]. However, some meta-analyses and randomized trials find no significant differences in medium- to long-term functional outcomes, implant survivorship, or quality of life compared to conventional techniques [13,35,38-40].

The translation of these technical improvements into long-term functional outcomes, implant survivorship, and cost-effectiveness remains debated, with many studies showing comparable medium- to long-term results between robotic and conventional approaches [13,31,35,39,41].

Robotic-Assisted Spinal Surgeries

Robotic surgery has rapidly changed orthopedic practice, primarily enhancing the accuracy of pedicle screw placement and supporting minimally invasive techniques. Since the first FDA approval in 2004, robotic-assisted spine surgery (RSS) has demonstrated high precision, reduced radiation exposure for surgical teams, and potential improvements in patient outcomes, especially in complex and deformity cases [42-46]. However, the literature also notes significant barriers, including high initial costs, a steep learning curve, and limited evidence on long-term clinical and cost-effectiveness outcomes [47-49].

Robotic-Assisted Trauma Surgery

The TiRobot System from China has pioneered robotic applications in orthopedic trauma, primarily focusing on percutaneous screw fixation for complex fractures. Clinical applications include pelvic ring fractures, proximal femur fractures, and intramedullary nail fixation for intertrochanteric fractures. Studies demonstrate significant benefits, including reduced operating time, decreased fluoroscopy use (70% reduction in radiation exposure), improved screw placement accuracy, and less intraoperative blood loss [16,25,50].

Expanding Indications: Trauma, Oncology, Complex Reconstructions

Robotic guidance in orthopedic trauma surgery demonstrates significantly shorter radiation exposure times compared to manual fluoroscopy, offering substantial safety advantages for healthcare providers and patients. A meta-analysis of 675 patients across 10 studies shows robust effect sizes supporting robotic system implementation in trauma applications [51].

**Figure 1:**
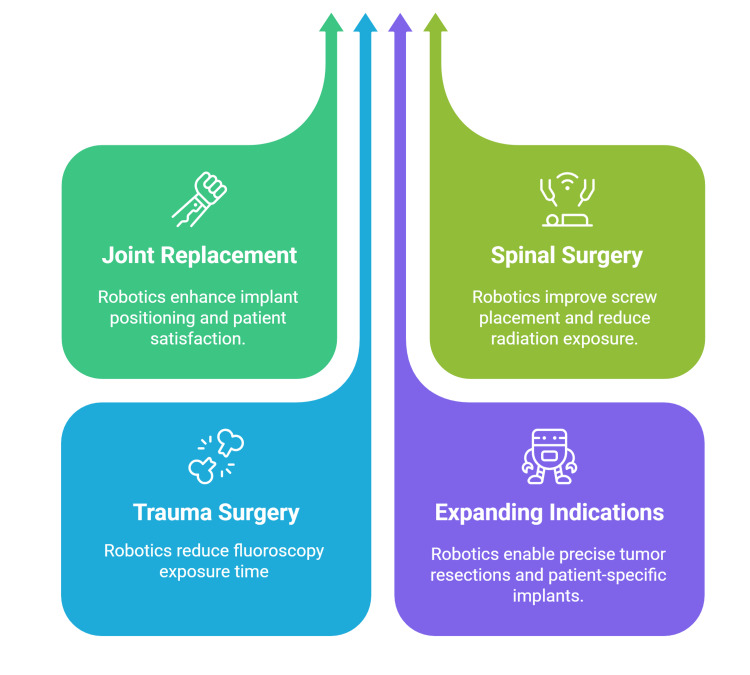
Clinical applications of robotic-assisted orthopedic surgery. Figure credit: Momen Abdelglil. Source: [38-53].

Orthopedic oncology increasingly utilizes robotic assistance for precise bone tumor resection and reconstruction procedures. Robotic systems enable accurate removal of complex bone volumes suitable for en bloc tumor removal, with precisely manufactured patient-specific implants achieving minimal gaps between cut surfaces. Electromagnetic navigation robot-assisted osteotomy for mandibular tumors demonstrates superior precision compared to conventional approaches, reducing excessive healthy tissue resection (Figure 1) [52,53].

Recent advances in robotic-assisted surgery in orthopedic practice

Artificial Intelligence Integration

Modern orthopedic robotic systems increasingly incorporate AI functionality to enhance performance through intelligent data analysis and real-time surgical optimization. AI algorithms assist surgeons by analyzing substantial datasets to optimize surgical processes, providing image recognition capabilities for lesion identification, and offering real-time guidance for prosthetic angle and position during implant placement procedures [54-56].

Advanced Imaging Technologies

Modern robotic orthopedic platforms make use of advanced imaging techniques, such as intraoperative cone-beam CT for tissue reconstruction and three-dimensional CT scans for preoperative planning. These systems provide dynamic safety control and real-time instrument deviation detection during surgical procedures by combining continuous optical tracking technologies with direct radiography image acquisition [57].

Optical Tracking Navigation Systems

Modern orthopedic robots use sophisticated optical tracking systems with in-house camera technologies that have a broad field of view for improved visibility of the probe and array. These systems, which use passive sphere markers and advanced optical navigation products to achieve sub-millimeter precision positioning during complex orthopedic surgical interventions, function noticeably faster than competing technologies. The TiRobot system employs an advanced optical tracking system and robotic arm technology, enabling sub-millimeter precision positioning in knee arthroplasty [25].

ExcelsiusGPS and CUVIS-Spine systems utilize real-time dynamic tracking of patients and surgical arms through optical trackers. These systems employ advanced optical navigation technologies with wide field-of-view cameras to ensure precise motion tracking and enhance surgical accuracy [58].

Recent investigations into optical tracking technologies in orthopedic surgery highlight the potential of mixed-reality headsets, such as Microsoft HoloLens 2, which use advanced depth sensors to achieve high-precision localization. Studies demonstrated that these systems can reliably track retro-reflective markers with clinically acceptable accuracy, supporting their use in intraoperative navigation. Frameworks like STTAR (Standardized Technical Testing of Augmented Reality) have been employed to benchmark performance, confirming that optical tracking can enhance spatial orientation, reduce registration errors, and improve workflow efficiency in orthopedic procedures [59].

Haptic Technology Integration

Robotic systems in orthopedics are generally classified as haptic (surgeon-guided) or autonomous. Haptic systems, which provide real-time force or tactile feedback, are more commonly used in joint reconstruction and spine surgery. Wearable and desktop haptic devices, as well as advanced sensors, are being developed to further enhance tactile realism and integration with surgical robots [27,60].

Despite clear benefits, technical challenges remain. These include the complexity of accurately sensing and reproducing forces, integrating tactile feedback into existing systems, and balancing cost-effectiveness. Most current systems provide only force feedback, with true tactile (texture, vibration) feedback still in early development. Widespread clinical adoption is also limited by cost, training requirements, and the need for robust validation in large-scale studies [27,61,62].

Intraoperative Imaging Advancements

Contemporary orthopedic robotic systems integrate real-time structured light imaging with CT guidance to reduce radiation exposure while providing superior three-dimensional surface reconstruction capabilities. These hybrid imaging approaches offer faster, higher-precision anatomical information compared to traditional CT-only systems, enabling real-time surgical feedback and enhanced instrument tracking accuracy [63].

Predictive Analytics Implementation

**Figure 2:**
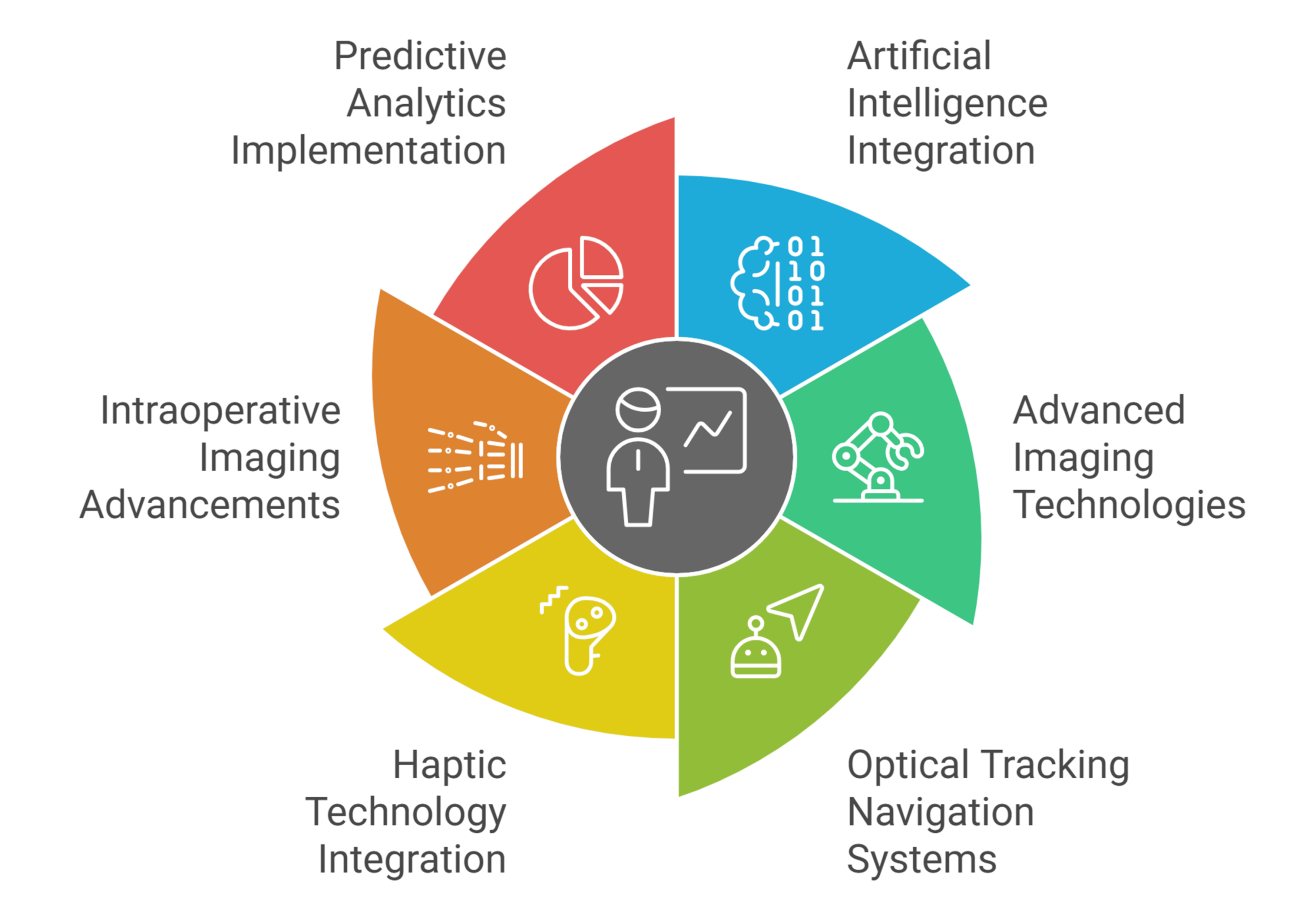
Recent advances in robotic-assisted surgery in orthopedic practice. Figure credit: Momen Abdelglil. Source: [54-64].

Advanced robotic platforms now incorporate machine learning algorithms for predictive outcome modeling, analyzing preoperative patient data, intraoperative metrics, and historical surgical databases to generate personalized treatment recommendations. These predictive systems help optimize implant selection, surgical approach customization, and complication risk stratification through comprehensive data-driven decision support mechanisms (Figure 2) [64].

Limitations, cost-effectiveness, and adoption barriers

Robotic systems are associated with very high upfront purchase expenses as well as ongoing maintenance costs, which can place a significant financial burden on healthcare institutions. In addition to the financial aspect, the adoption of these systems is also linked to increased operative times, particularly during the initial learning phase, when surgeons are still becoming familiar with the technology and its workflow. Furthermore, the effective use of robotic platforms requires extensive and specialized training for surgeons, demanding both time and resources to ensure proficiency and patient safety [6,27,28,31,65,66].

The cost-effectiveness of robotic surgery is generally realized in large, high-volume centers where a significant number of procedures are performed regularly. These institutions can distribute the high initial investment and ongoing maintenance costs of robotic platforms over a greater number of surgeries, which reduces the financial burden per case. Additionally, maintaining low revision and complication rates in such centers further contributes to cost savings, as fewer resources are required for corrective procedures or extended hospital stays [31,67].

The adoption of robotic surgery is influenced not only by clinical outcomes but also by nonclinical factors such as marketing strategies, administrative pressure to remain competitive, and patient perceptions of technological superiority. At the same time, successful implementation depends on ensuring that patients receive accurate education about the benefits and limitations of robotic procedures, while surgeons acquire sufficient training and experience to use these systems safely and effectively [68,69].

Future directions

Integration of AI and Machine Learning for Surgical Planning and Execution

Machine learning algorithms are revolutionizing orthopedic robotic surgery through enhanced preoperative planning and real-time decision-making. Recent research demonstrates that convolutional neural networks achieve high accuracy in fracture detection and osteoarthritis grading, enabling precise prosthetic sizing and complication minimization. Advanced AI-powered systems now optimize preoperative planning by predicting joint centers and identifying complications using multimodal data integration [70,71].

Deep learning models have transformed traditional manual processes in orthopedic surgery, providing automated hip osteoarthritis diagnosis and personalized risk stratification. These AI applications offer innovative solutions for implant positioning precision and patient-specific treatment protocols. Machine learning's predictive capabilities significantly outperform traditional scoring systems, particularly in mortality and complication prediction for complex orthopedic procedures [71].

Advances in Augmented Reality and Real-Time Feedback

Mixed reality technology addresses critical limitations in orthopedic oncology by providing real-time visual feedback during surgical procedures. Current navigation systems require surgeons to shift attention from operative fields to monitors, while mixed reality overlays preoperative images directly onto surgical sites [72].

Augmented reality has gained substantial traction in orthopedic surgery for intraoperative guidance and decision-making across procedures, including tumor resection, fracture fixation, and joint arthroplasty. Real-time imaging data integration allows surgeons to visualize computer-processed information seamlessly combined with live surgical views. Advanced hybrid operating rooms now incorporate mixed reality glasses alongside robotic arms and navigation systems for enhanced precision [73,74].

Recent developments in machine learning vision models analyze surgical videos in real-time, detecting defects and enabling automatic parameter adjustments without human intervention. These systems provide surgeons with immediate feedback through depth cameras and specialized computer vision setups designed for close-range monitoring. Clinical deployment of real-time surgical guidance achieves prediction streams exceeding 60 fps with maximum delays under 70 milliseconds [75].

Development of More Autonomous and Tactile Robotic Systems

Autonomous robotic surgery represents the integration of varying degrees of independence for surgical procedure execution, enabled by AI and machine learning advances. Contemporary systems employ imitation learning combined with constraint optimization to enhance automation in procedures like pedicle screw implantation while ensuring reliability and safety. Current research demonstrates distance errors of 0.738 ± 0.080 mm in autonomous orthopedic procedures using advanced motion planning algorithms [76].

**Figure 3:**
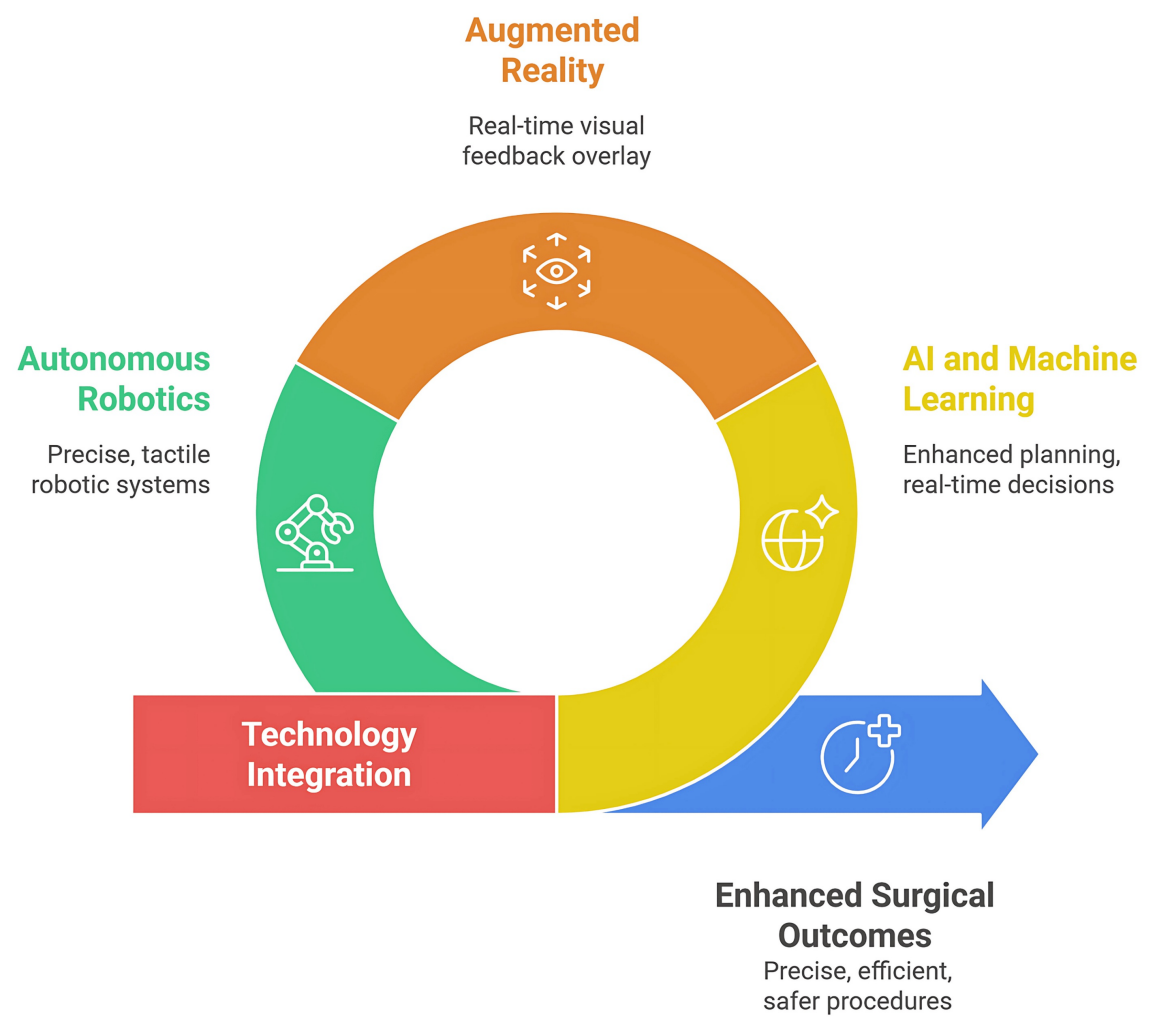
Recent innovations and future advances in orthopedic surgery. Figure Credit: Momen Abdelglil. Source: [71-77].

Haptic robot-assisted technology shows significant potential for enhancing primary bone tumor resection accuracy compared to manual techniques. Early research indicates tactile feedback systems improve surgical precision in wide resection procedures, though most systems remain in experimental phases. Force feedback mechanisms are being integrated into robotic platforms to provide surgeons with enhanced sensitivity during delicate orthopedic manipulations (Figure 3) [8,77].

Modern robotic systems incorporate motion scaling, tremor elimination, and enhanced depth perception capabilities, achieving 100% patency rates for microsurgical procedures. These advances enable smaller incisions and faster recovery with reduced complications in complex reconstructive surgeries. Contemporary platforms like the Symani Surgical System demonstrate successful implementation of tactile feedback in upper extremity reconstructions with minimal operative time increases [78].

## Conclusions

Robotic-assisted surgery is transforming orthopedic practice by enhancing precision, reproducibility, and short-term outcomes across joint replacement, spine, trauma, and oncologic procedures. Advances in imaging, AI, navigation, and haptic feedback are broadening its applications, though long-term benefits and cost-effectiveness are still under evaluation.

Despite challenges such as high costs, training demands, and limited accessibility, the future of robotic surgery in orthopedics is promising. Continued integration of AI, augmented reality, and automation is expected to further personalize care, but large-scale studies and standardized training are essential for its sustainable and widespread adoption.
